# Description of a new species of *Aleuroclava* Singh, 1931 (Hemiptera, Aleyrodidae) infesting *Schima superba* from China

**DOI:** 10.3897/zookeys.991.47725

**Published:** 2020-11-11

**Authors:** Ji-Rui Wang, Zhi-Hong Xu, Guo-Xin Zhou

**Affiliations:** 1 School of Agricultural & Food Science, Zhejiang Agriculture & Forestry University, Linan, Zhejiang 311300, China Zhejiang Agriculture & Forestry University Linan China

**Keywords:** *Aleuroclava schimae* sp. nov., instar, morphology, new species, puparia, taxonomy, whitefly

## Abstract

A new whitefly species, *Aleuroclava schimae* Wang, **sp. nov.** infesting leaves of *Schima superba* (Parietales, Theaceae) is described and illustrated from Zhejiang, China. Puparia of the new species are elliptical, broad at the transverse molting suture region and broadly truncate posteriorly. Thoracic and caudal tracheal pores are discernible. In life, the puparia are covered by a thin layer of white wax.

## Introduction

The whitefly genus *Aleuroclava* Singh, 1931 is represented by 124 species worldwide, of which 38 species are from China (Evans 2007; Wang et al. 2014; Wang and Du 2016), including *A. schimae* sp. nov.. *Aleuroclava* species occur predominantly in the Oriental and Austro-Oriental Regions and feed on a wide range of host plants (Evans 2007). Wang and Du (2016) provided a diagnostic key to *Aleuroclava* species including those of Hong Kong and Taiwan. *Aleuroclava schimae* sp. nov. found densely infesting leaves of *Schima superba* Gardner & Champ. at Thousand Island Lake (TIL), Gutianshan Nature Reserve, Shuangxikou village, Zhejiang, China is described herein. Morphological characteristics of puparia and immatures of the new species are described with images of habitus, holotype, line drawings and SEM images.

*Schima superba* (Parietales, Theaceae) is an economically and ecologically important woody tree of China. It is a dominant tree species in the subtropical evergreen broad-leaved forests of southern China (Zhang et al. 2019), and commercially used for timber, furniture and construction purposes, and also as fire breaks to prevent forest fires (Yang et al. 2017).

## Material and methods

Puparia of the new species were collected on leaves of *Schima superba* from Zhejiang, Thousand Island Lake (hereafter TIL) and Gutianshan Nature Reserve, Shuangxikou village, China. No adult emergence was noticed during rearing of puparia for two weeks. Puparia were mounted following Dubey and David (2012). The terminology for morphological structures follows Bink-Moenen (1983), Martin (1985) and Gill (1990). Habitus images were taken using a digital camera Canon IXUS 105 and a camera DFC 290 (Leica, Wetzlar, Germany) attached to a Leica stereomicroscope M 125 (Leica, Wetzlar, Germany). Puparial measurements and microphotographs were taken using a compound microscope (Carl Zeiss, Gottingen, Germany) from Zhejiang Agriculture and Forestry University (ZAFU). The scanning electron microscope images were taken by Hitachi TM-1000 Scanning Electron Microscope (Hitachi, Japan) from the Center of Electron Microscopy, Zhejiang University (Life Sciences Division). Adobe Photoshop 7 software was used for figure preparation. The holotype is deposited in the Insect Collections of Zhejiang Agriculture and Forestry University, Lin’an, China (ZAFU). One paratype will be deposited in the Shanghai Entomological Museum, Chinese Academy of Sciences (SEM-CAS) and the remainder in ZAFU.

## Taxonomy

### Aleuroclava

Taxon classificationAnimaliaHemipteraAleyrodidae

Singh

58CF53A1-471E-576A-B53E-46F60A0EA5BC

#### Diagnosis.

Puparia small in size, elliptical or subelliptical. Margin with one row of teeth. Submarginal area not separated from dorsal disc, with papillae-like markings in some species, dorsum generally with tubercles. Thoracic tracheal folds and pores not discernible; caudal furrow and pore distinct. Vasiform orifice generally notched posteriorly; operculum cordate, nearly filling orifice; lingual hidden.

**Figures 1–2.**
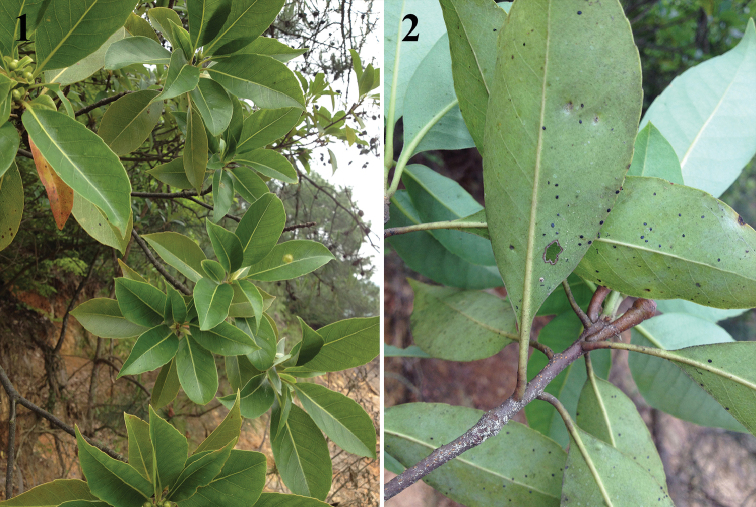
Leaves of host plant *Schima superba* infested by *A. schimae* sp. nov.

### Aleuroclava schimae

Taxon classificationAnimaliaHemipteraAleyrodidae

Wang sp. nov.

04C1701C-2884-588C-88C1-8724F5D287F8

http://zoobank.org/2A18B6BA-16DC-41CD-834F-FCE9AA22EDB5

Figs 3–8 , 9–12 , 13–15 , 16–18

#### Type material.

***Holotype***: China, Zhejiang, Zhejiang, Chun’an, Thousand Island Lake, 1 puparium on slide, on *Schima superba*, 6. vi. 2016, 29°31.21'N, 118°52.41'E, leg. JR Wang. Deposited in the Insect Collections of ZAFU, Lin’an, China. ***Paratypes***: Fifty-six, of which 30 puparia on 21 slides, data same as for holotype; Gutianshan Nature Reserve, Zhejiang, Kaihua, 15 puparia on 12 slides, on *Schima superba*, 28.xiii.2018, 29°15.12'N, 118°06.42'E, leg. AQ Dai; Zhejiang, Jiangshan, Shuangxikou village, 11 puparia on 10 slides, 30. xiii. 2018, 28°23.12'N, 118°41.15'E, leg. AQ Dai. (SEM-CAS 1 paratype, ZAFU 55 paratypes and dry collection).

**Figures 3–8.**
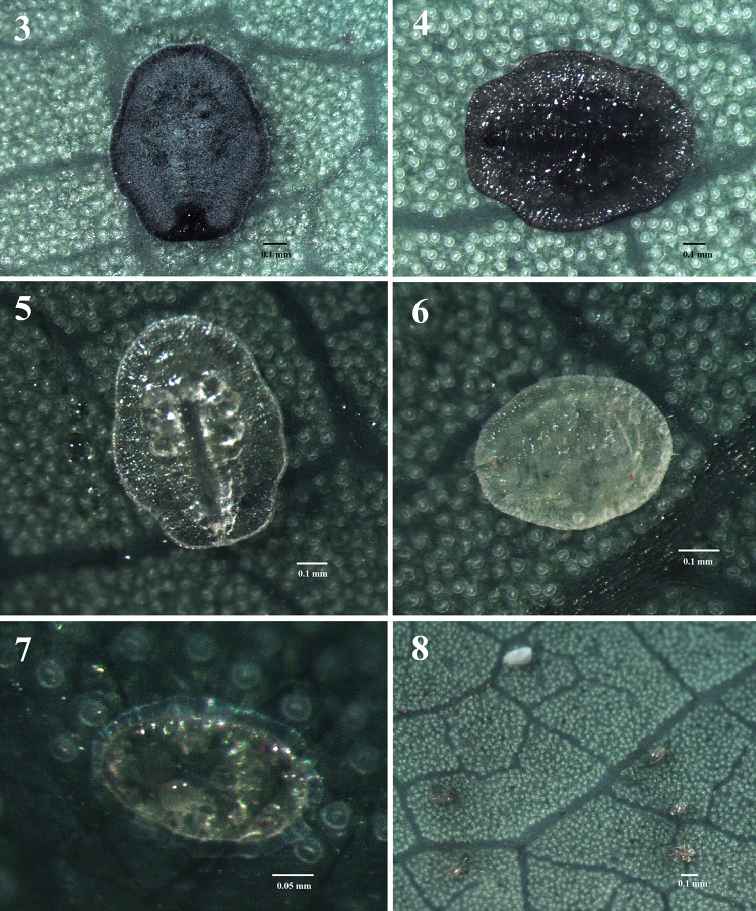
Habitus, developmental stages of *A. schimae* sp. nov., on *S. superba*. **3, 4** puparium, late stage **5** puparium, early stage **6** third instar **7** second instar **8** eggs.

#### Description.

***Egg*** (Fig. 8). Fusiform; yellowish, gradually becoming dark brown over time; about 152 µm long, 69 µm wide, found deposited randomly on lower surface of leaves.

***Puparium*.** Covered by a thin layer of white wax (Fig. 3); puparium in early stage white (Fig. 5), gradually turns black (Figs 3, 4), about 926–1120 µm long, 763–832 µm wide; elliptical, broadest at the transverse molting suture region; posteriorly horizontal.

**Figures 9–12.**
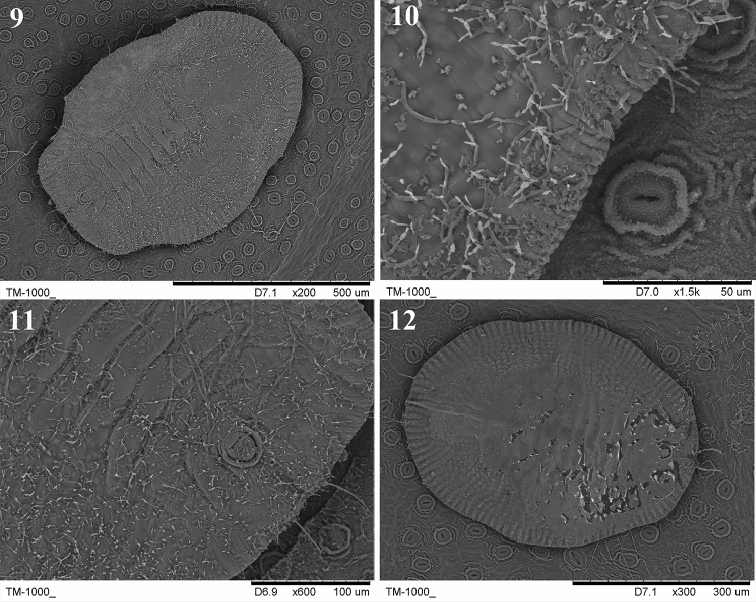
Scanning electron microscope (SEM) photographs of *A. schimae* sp. nov. **9** puparium, dorsal view **10** margin **11** vasiform orifice and operculum **12** third instar nymph.

**Figures 13–15.**
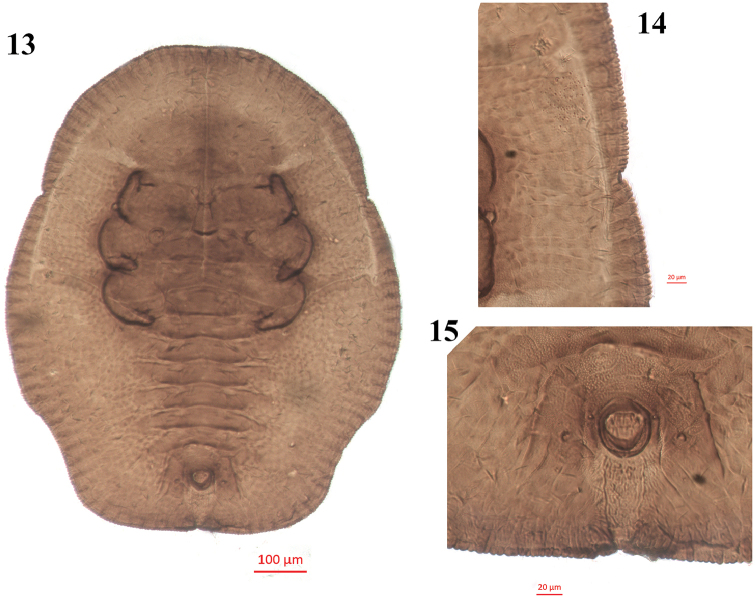
*Aleuroclava schimae*, sp. nov., slide mounted specimen. **13** puparium, dorsal view **14** margin **15** vasiform orifice and operculum.

**Figures 16–18.**
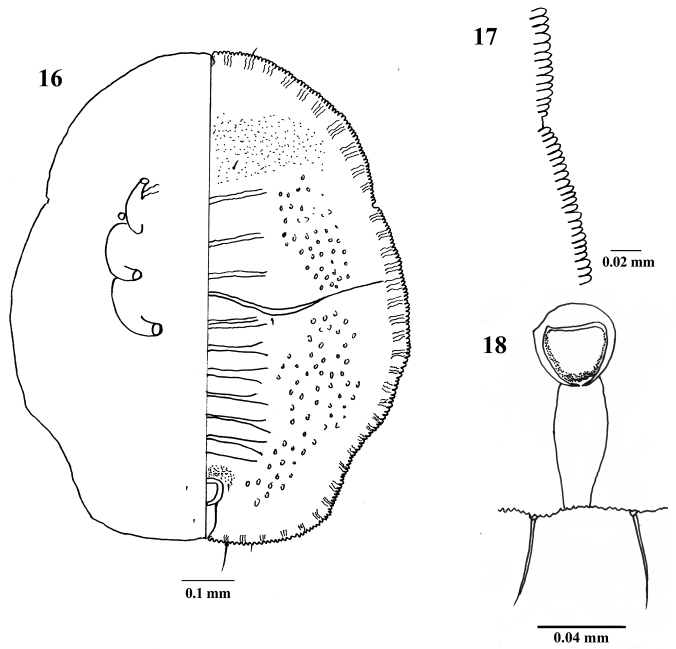
*Aleuroclava schimae* sp. nov., holotype puparium, China, Zhejiang. **16** puparium, dorsal (right) and ventral (left) views **17** margin **18** vasiform orifice.

***Margin*** (Figs 10, 14, 17). Crenulate, 0.1 mm wide, 23–28 crenulations. Paired anterior and posterior marginal setae 27 and 16 µm long, respectively. Thoracic tracheal pore area slightly recessed and emarginated at margin. ***Dorsum*.** Almost flat, slightly raised on abdomen; without tubercles. Submargin demarcated from the dorsal disc by a faint line. Longitudinal moulting suture reaching anterior margin and the transverse moulting suture reaching submargin (Figs 13, 16). Thoracic and abdominal segment sutures well defined. Middle length of abdominal segment I 53 µm; segment II 47 µm; segments III–VI subequal, 37 µm; segment VII 28 µm long. Geminate pores present (Figs 9, 13, 16). ***Chaetotaxy*.** Cephalic, first, eighth abdominal and caudal setae 9, 13, 4 and 47 µm long, respectively. Eighth abdominal setae located below the base of orifice. Caudal furrow 68 µm long. ***Vasiform orifice*** (Figs 11, 15, 18). Cordate to subcircular, slightly longer than wide, 48 µm long, 44 µm wide, lateral margins rounded, basal ends being curved to meet basal margin; operculum cordate, 33 µm long, 29 µm wide, almost covering the orifice and obscuring the lingula. ***Venter*.** Thoracic and caudal tracheal folds discernible (Fig. 16). Ventral abdominal setae placed on anterior to vasiform orifice, 6 µm long, 49 µm apart. Antennae extending near the base of prolegs. ***Third instar nymph*** (Figs 6, 12). Light yellow, body transparent, elliptical, about 720 µm long, 540 µm wide; eye spots obvious. ***Second instar nymph*** (Fig. 7): yellowish, elongate-elliptical, about 290 µm long, 170 µm wide; transparent wax secretion along the body margin, about 11 µm wide; eye spots red.

#### Host plant.

*Schima superba* Gardner & Champ (Parietales, Theaceae) (Figs 1, 2).

#### Distribution.

China: Zhejiang.

#### Biology.

Puparia were found on the lower surface of leaves; 10–40 per leaf (Figs 2–4); covered by a thin layer of white wax (Fig. 5). Exuviae of previous instars were present. No parasitoids and ants were observed.

#### Etymology.

The species is named after the host plant, *Schima superba*.

#### Remarks.

Puparia of the new species are elliptical in outline, broad at the transverse moulting suture region, truncate posteriorly (Figs 3, 4, 9, 13, 16), thoracic tracheal pores recessed, emarginated (Figs 10, 14, 17), transverse moulting suture reaching submargin (Figs 13, 16), and vasiform orifice cordate to subcircular, slightly longer than wide.

Puparium of *A. schimae* sp. nov. resembles that of *A. tianmuensis* in body shape, size and colour, but differs in having horizontal posterior end (curved in *A. tianmuensis*), and in lacking median tubercles on abdominal segments II-VI. It differs from *Aleuroclava similis* (Takahashi) in colour of puparium, and from *A. trivandricus* Dubey & Sundararaj in colour and thoracic tracheal pores not deeply inset at the margin. It also differs from *A. hikosanensis* (Takahashi) from the characteristic of the median area of each abdominal segment.

## Supplementary Material

XML Treatment for Aleuroclava

XML Treatment for Aleuroclava schimae
